# Supplementary figures and images for: SpatialKNifeY (SKNY): Extending from spatial domain to surrounding area to identify microenvironment features with single-cell spatial omics data
Source: PLoS Comput Biol. 2025 Feb 18;21(2):e1012854. doi: 10.1371/journal.pcbi.1012854 (PMC11849985; doi:10.1371/journal.pcbi.1012854)

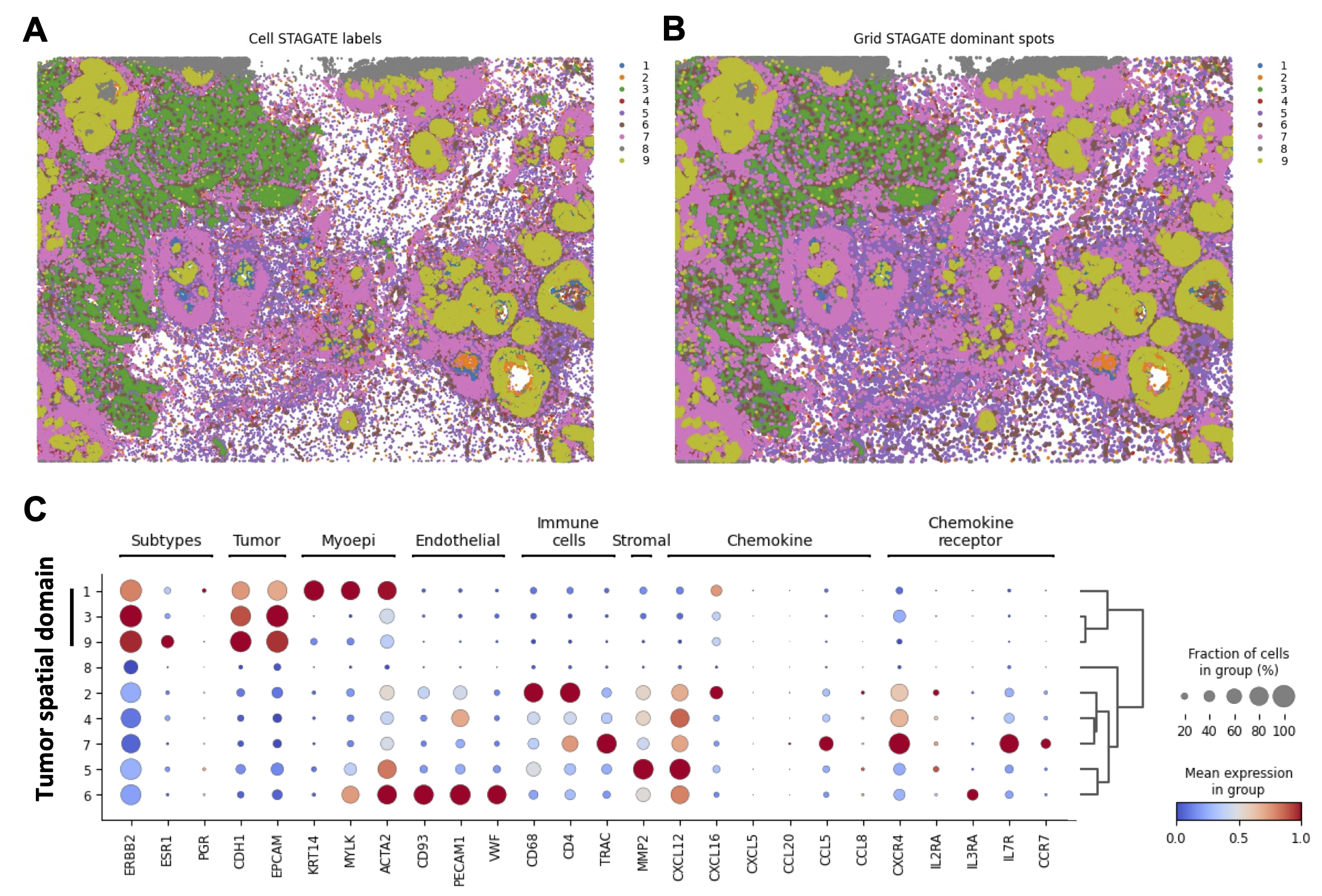

Supplement: S1 Fig — Spatial distribution of each cluster by STAGATE algorithm at (A) single-cell level and (B) grid level. (C) Dotplot showing markers of cell types and expression patterns of genes associated with tumor subtypes. Clusters 1, 3, and 9 correspond to the tumor spatial domain. (TIF) [file pcbi.1012854.s001.tif]

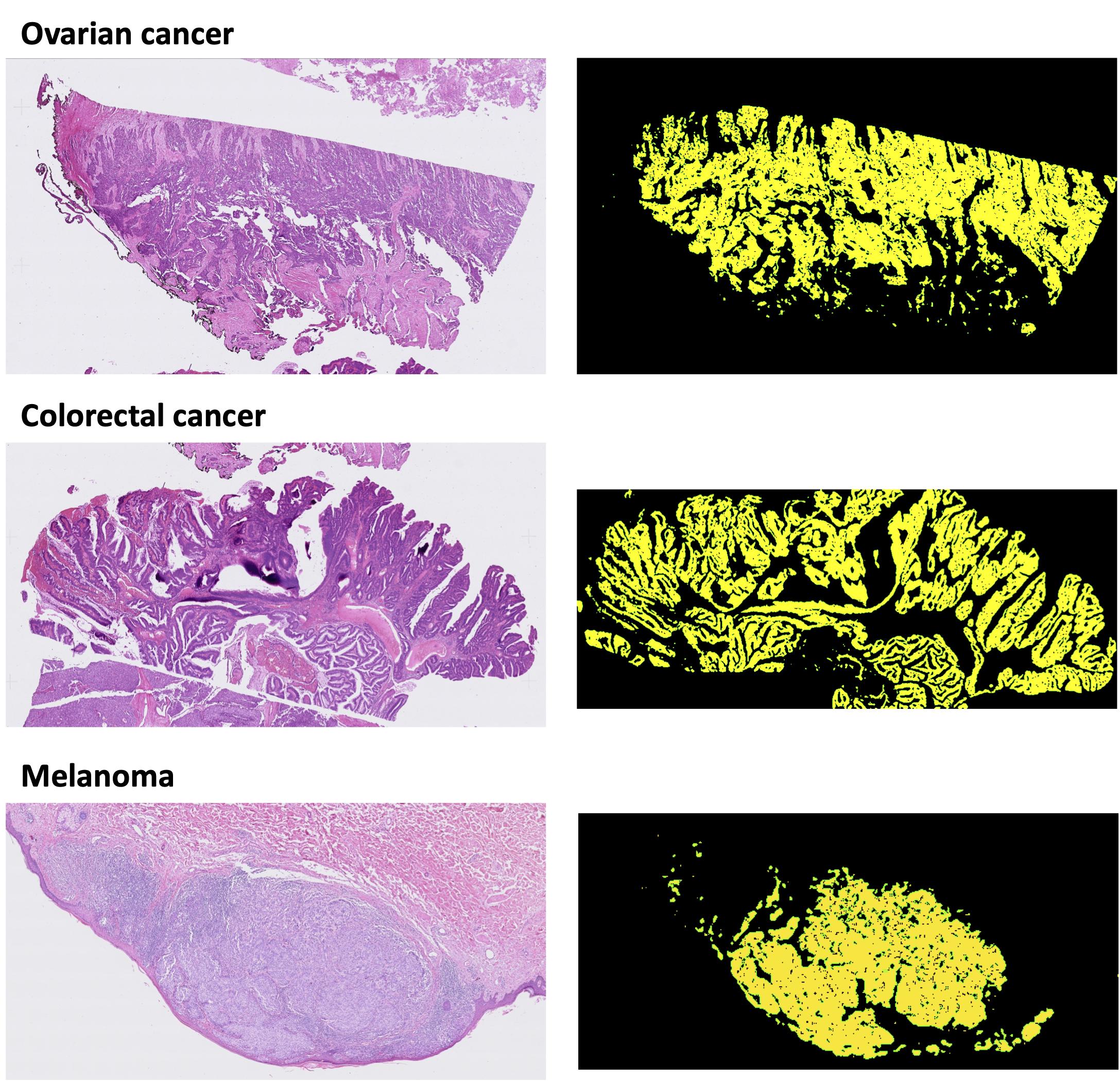

Supplement: S2 Fig — H&E staining images and detected spatial domains of ovarian cancer, colorectal cancer, and melanoma. The yellow and green colors indicate spatial domains and the boundary, respectively. (TIF) [file pcbi.1012854.s002.tif]

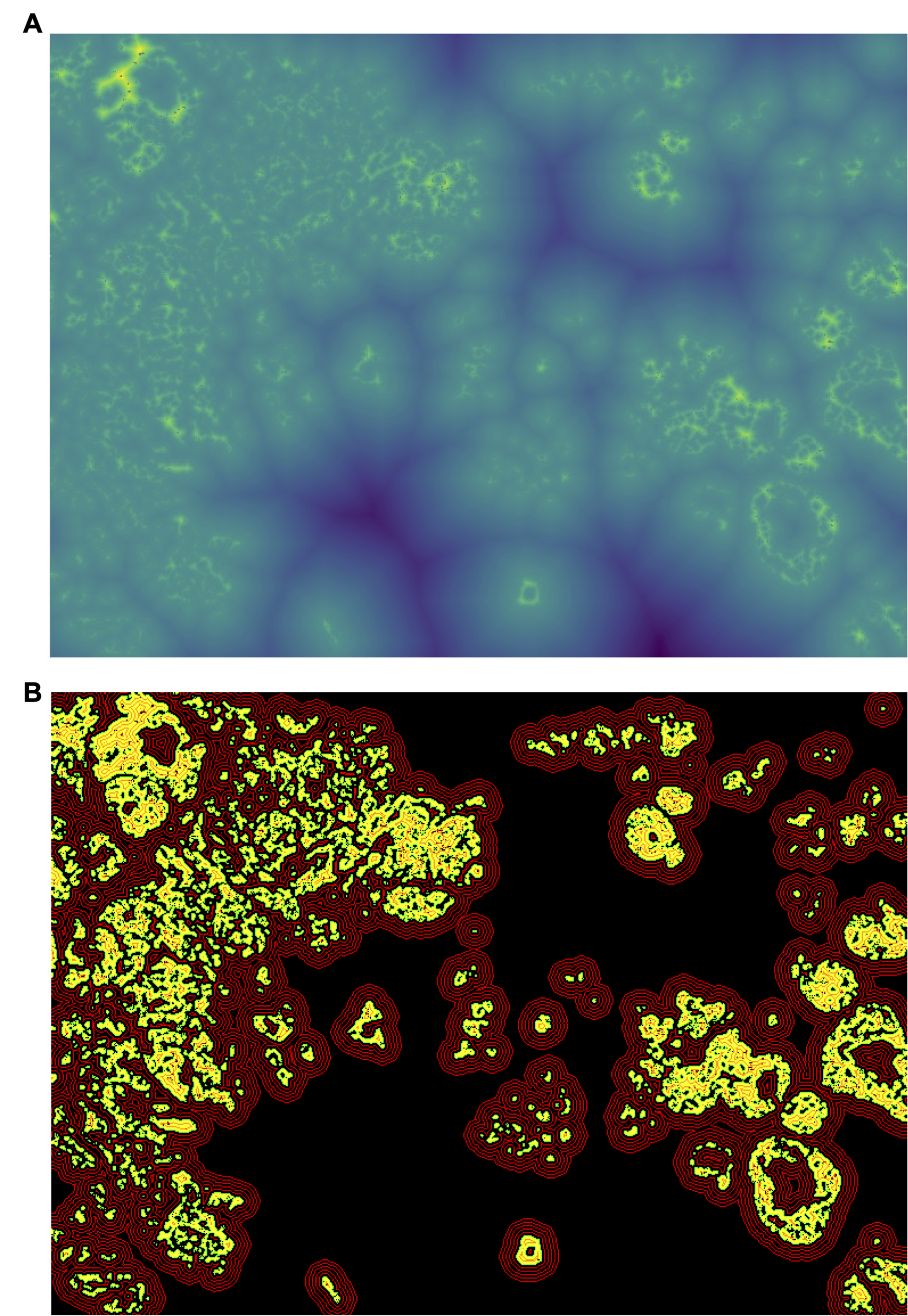

Supplement: S3 Fig — (A) Heatmap indicating distance from surfaces of spatial domains. (B) The red contour lines indicate distance from the surface of spatial domains at the 30-μm interval. (TIF) [file pcbi.1012854.s003.tif]

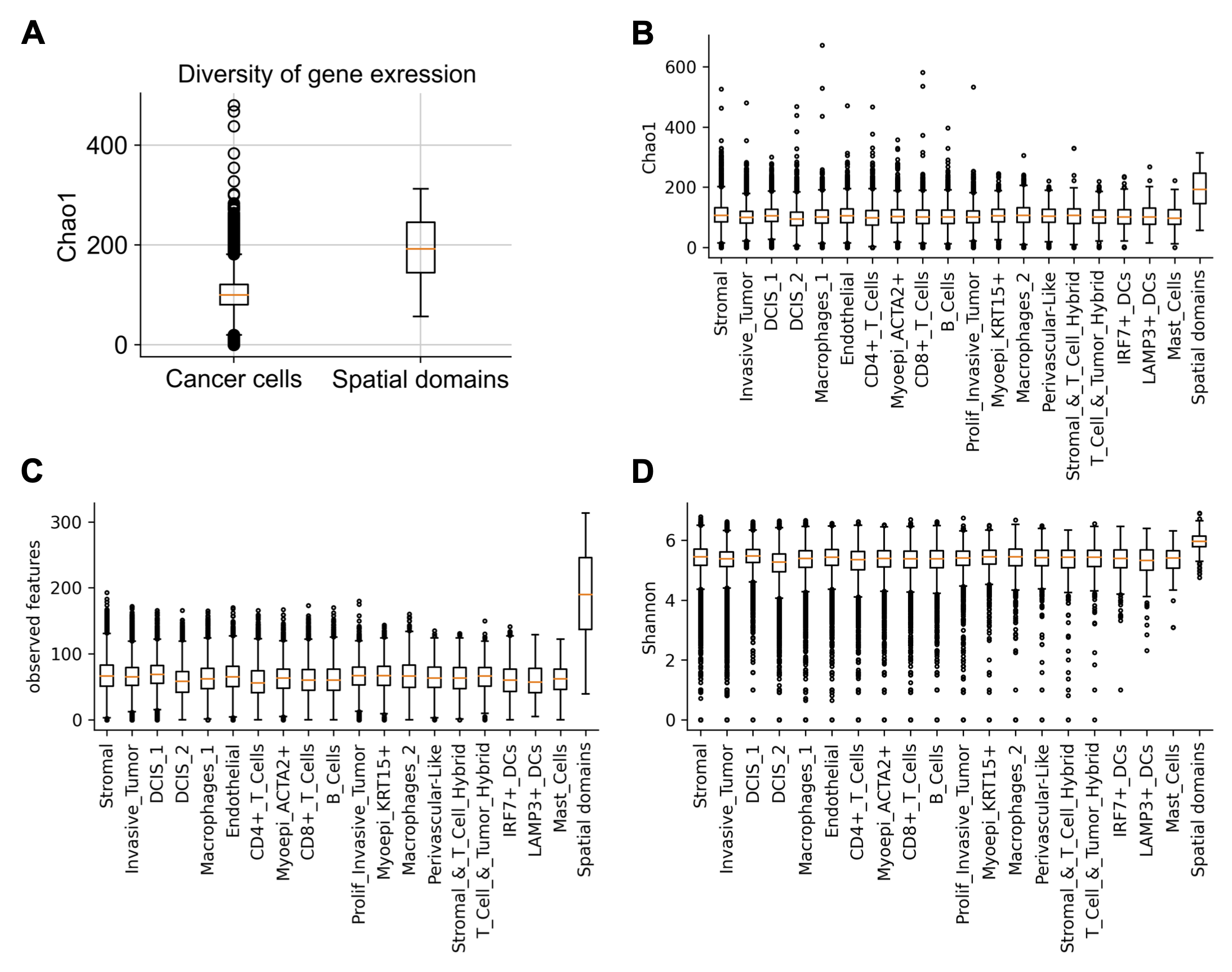

Supplement: S4 Fig — (A) Box plot of alpha-diversity index (Chao1) of expression between cancer cells and spatial domains. Box plot of (B) Chao1, (C) observed features, and (D) Shannon of expression among cells annotated by Janesick et al. and spatial domains. (TIF) [file pcbi.1012854.s004.tif]

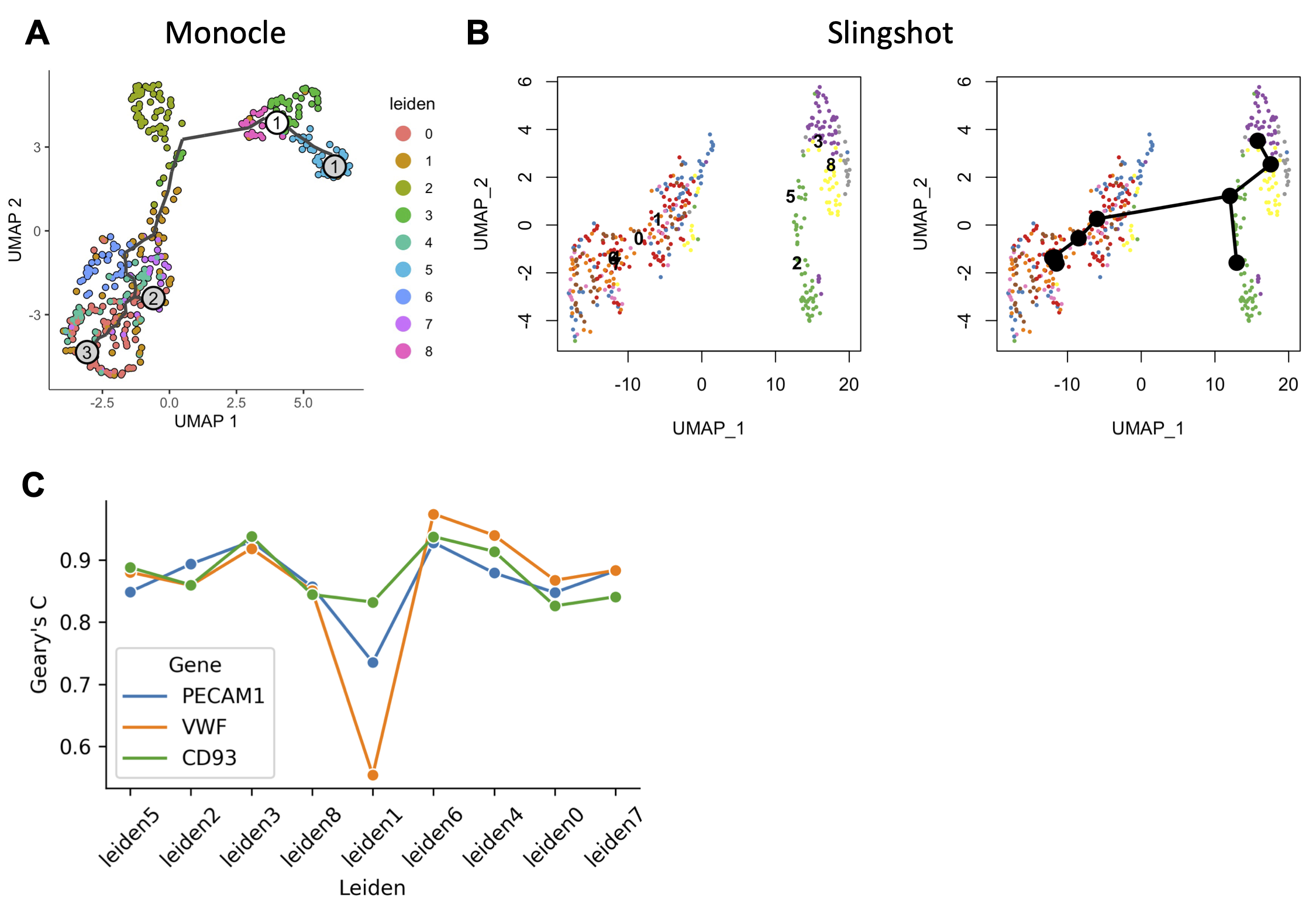

Supplement: S5 Fig — (A) The plot shows the Uniform Manifold Approximation and Projection (UMAP) representation of spatial domains, colored by their Leiden cluster assignments, overlaid with the trajectories predicted by the Monocle algorithm. The root point of the trajectory is labeled by the white node, and the branch points are labeled by the gray node. (B) The plot shows the UMAP representation of spatial domains, overlaid by their Leiden cluster assignments (left), overlaid with the trajectories predicted by the Slingshot algorithm (right). (C) Line plot depicting the relationship between Leiden clusters and Geary’s C for the genes PECAM1 (blue), VWF (orange), and CD93 (green). (TIF) [file pcbi.1012854.s005.tif]

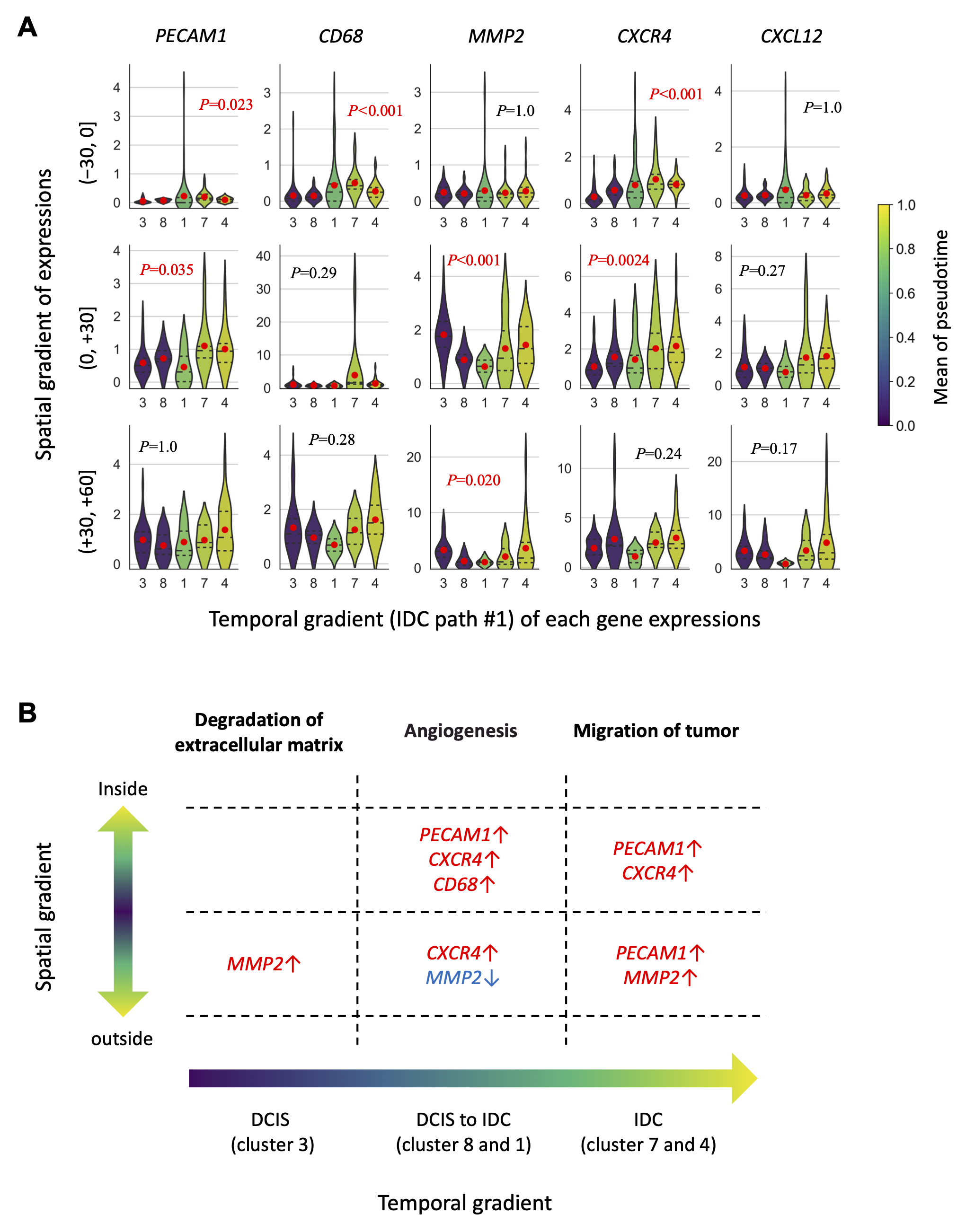

Supplement: S6 Fig — (A) Violin plots showing marker expressions of PECAM1, CD68, MMP2, CXCR4, and CXCL12 on the estimated trajectory path (IDC path #1) in the (−30, 0], (0, +30], and (+30, +60] sections. The color scale indicates the mean of pseudotimes in each cluster. The annotated values represent P values of the significance test. Red dots in the Figs indicate the mean the gene expression level. (B) Summary of the gene expression dynamics. Red and blue colors indicate the overrepresentation and underrepresentation of the gene expressions. (TIF) [file pcbi.1012854.s006.tif]

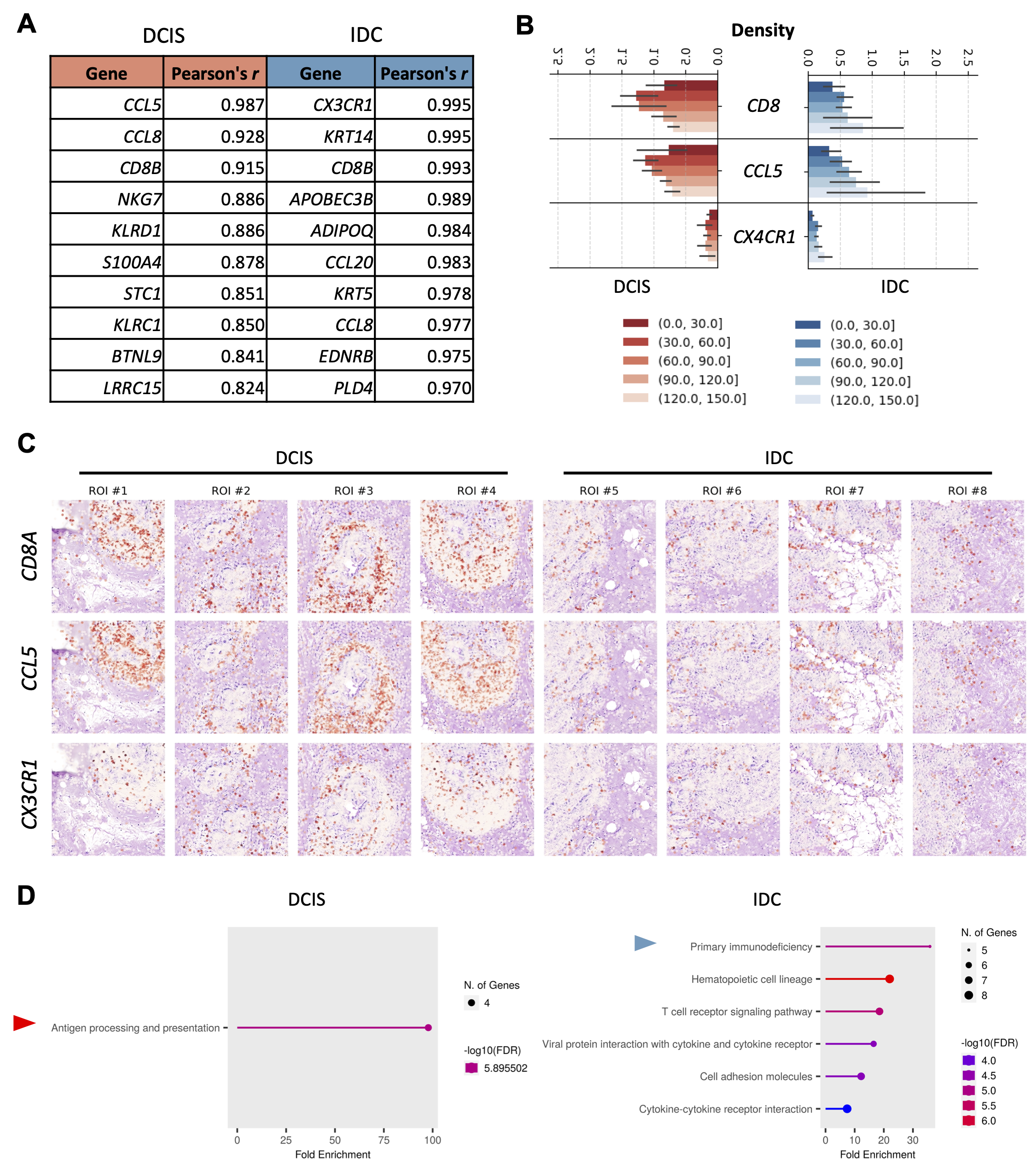

Supplement: S7 Fig — (A) Top 10 genes with correlation coefficients with CD8A of DCIS or IDC. (B) Bar plot shows the expression density of CD8A, CCL5, and CX3CR1 stratified by section. The red series indicates DCIS, and the blue series indicates IDC. The respective color gradients indicate each stratified interval. Error bars represent 95% confidence intervals (CI). (C) Spatial expression distribution of CD8A, CCL5, and CX3CR1 in each ROI. (D) Lollipop plot shows significantly enriched KEGG pathways. The x-axis denotes fold enrichment, plot size represents the number of genes, and the color represents −log10(FDR). (TIF) [file pcbi.1012854.s007.tif]

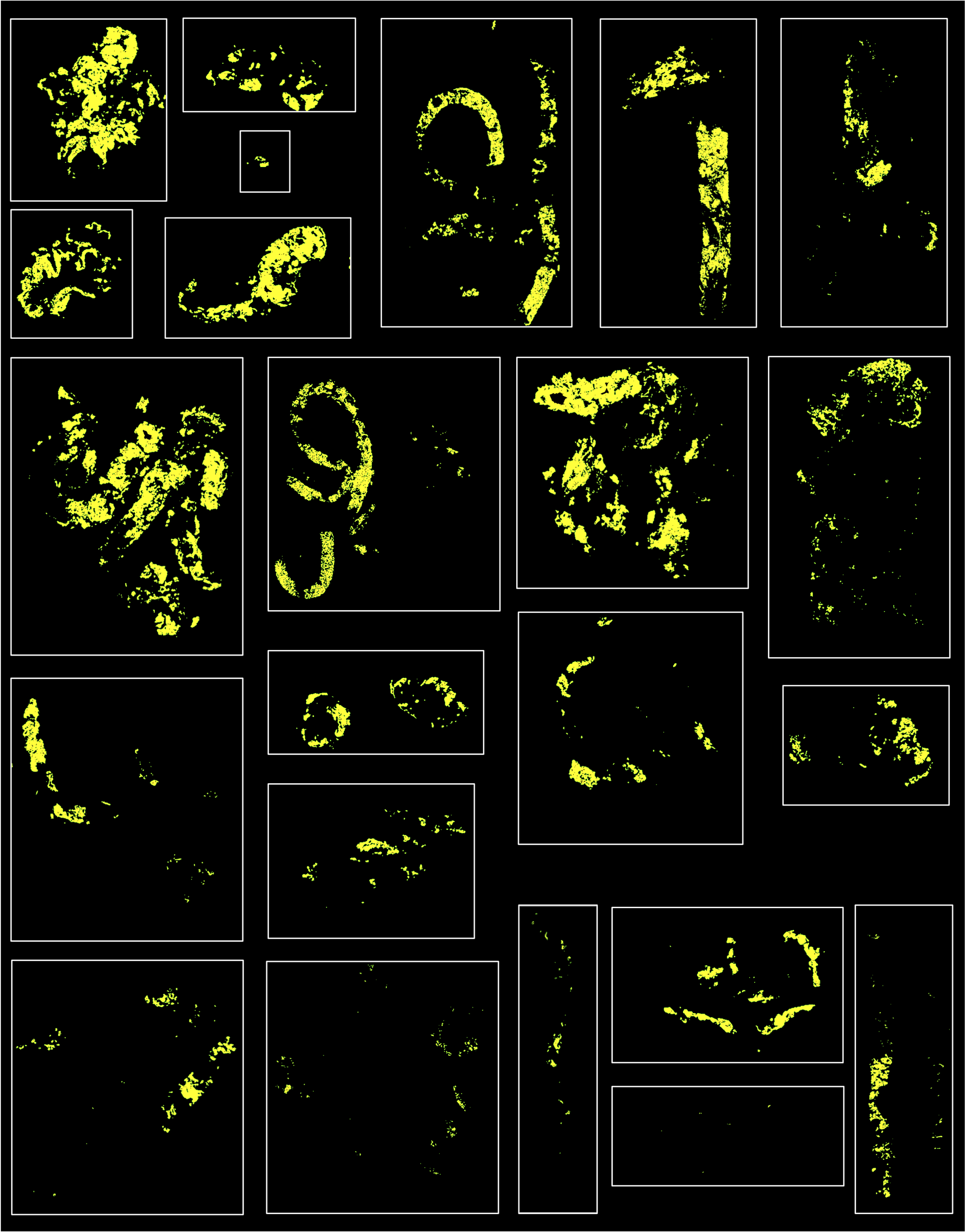

Supplement: S8 Fig — Spatial domains of metastatic colorectal cancer. The yellow and green colors indicate spatial domains and the boundary, respectively. (TIF) [file pcbi.1012854.s008.tif]

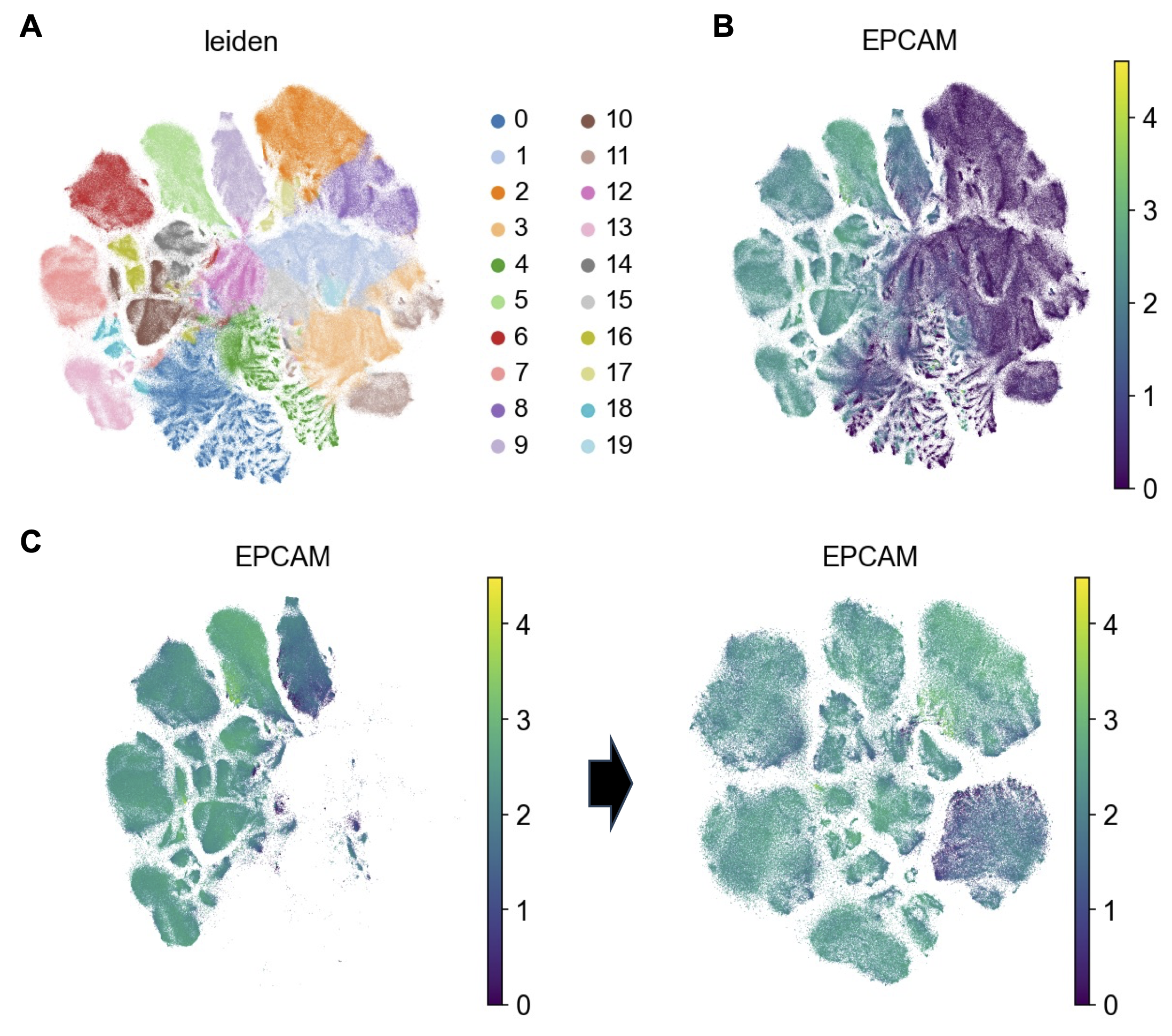

Supplement: S9 Fig — (A) t-Distributed stochastic neighbor embedding (tSNE) plot of 1,133,514 cancer cells. The plot is colored according to the clusters determined by the leiden algorithm. (B) tSNE from A colored by EPCAM expression. (C) Extraction of clusters with high EPCAM and rearrangement of tSNE space. (TIF) [file pcbi.1012854.s009.tif]

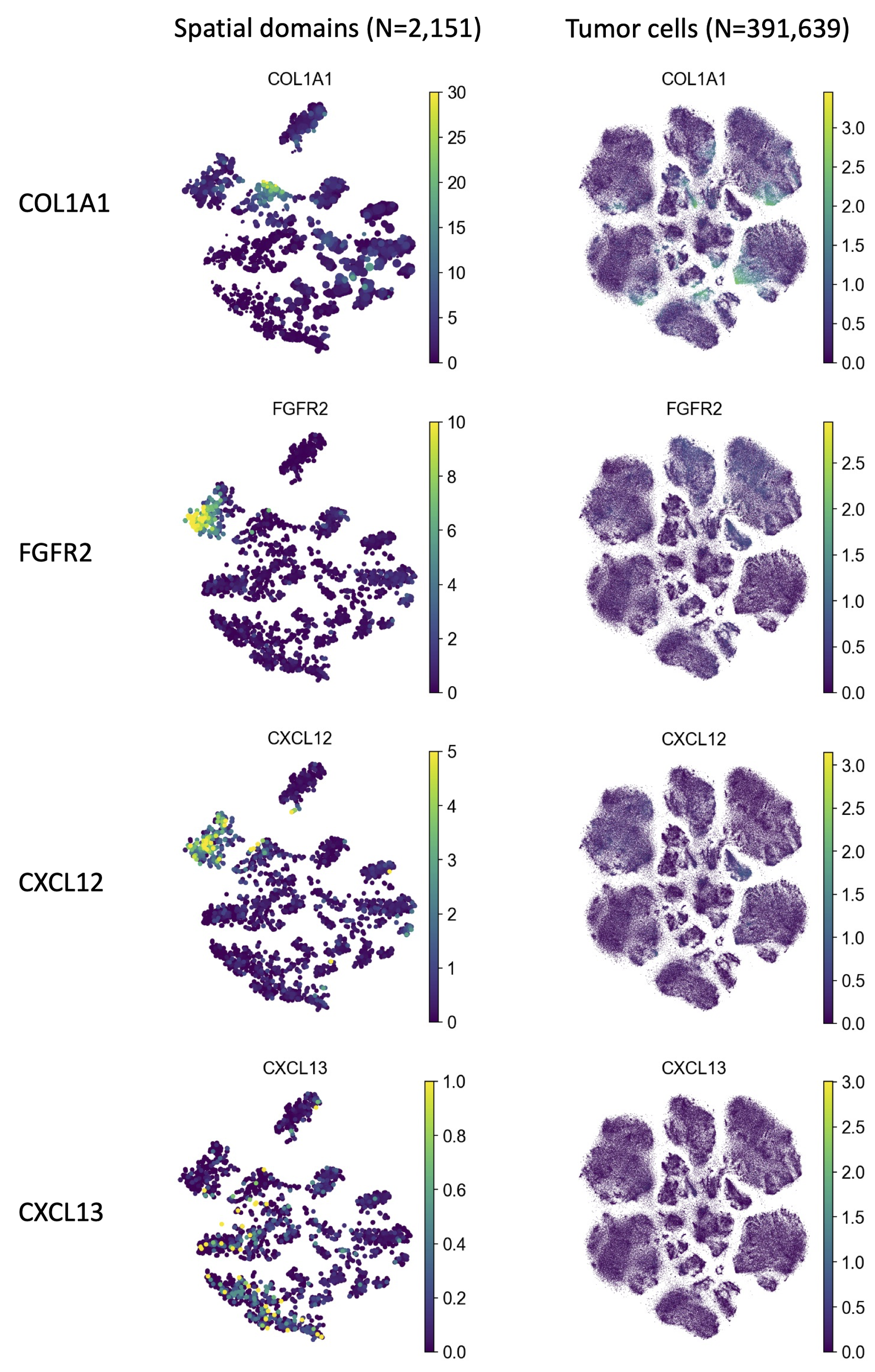

Supplement: S10 Fig — The plots are colored according to the expression of COL1A1, FGFR2, CXCL12, and CXCL13. (TIF) [file pcbi.1012854.s010.tif]

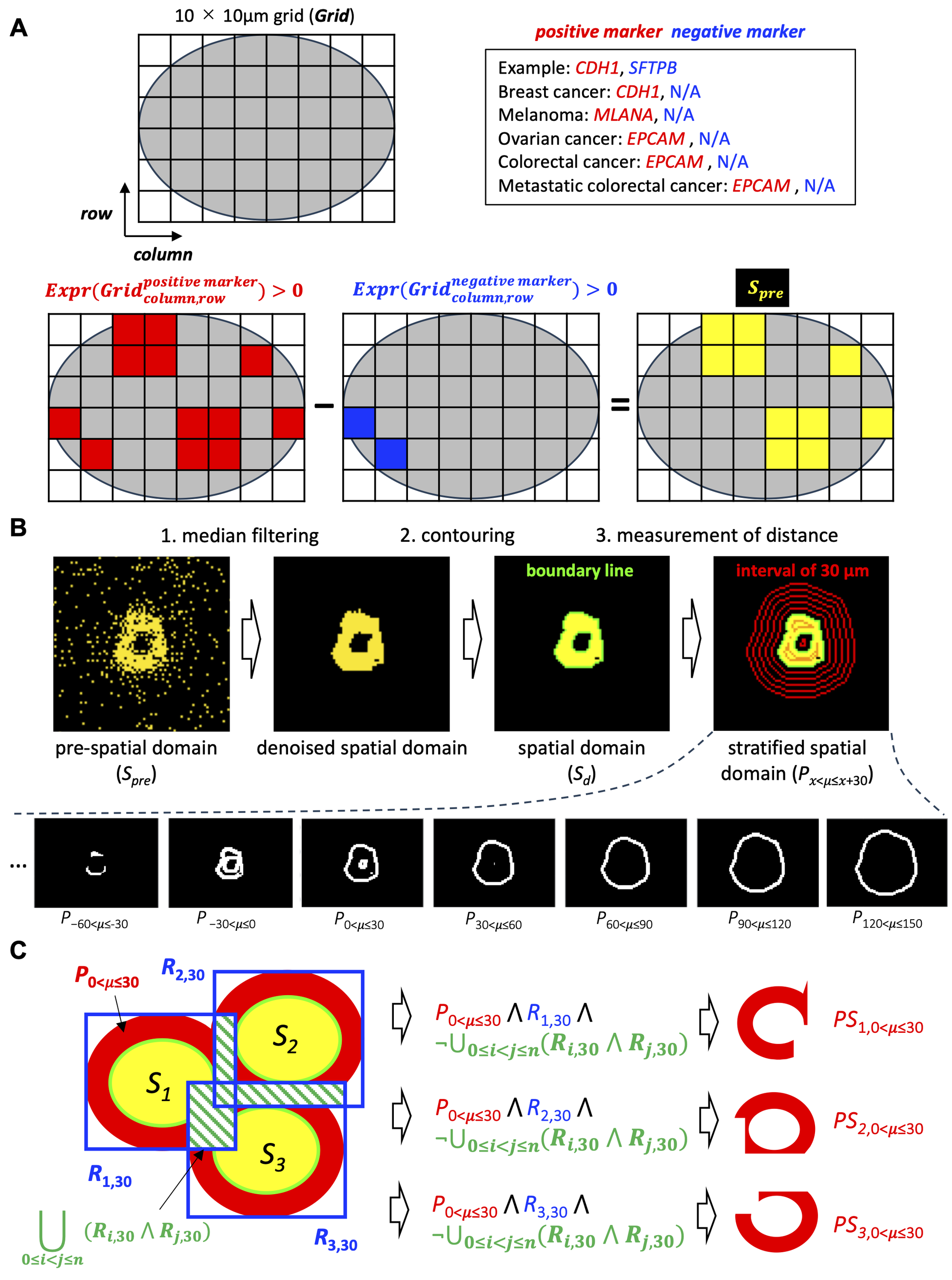

Supplement: S11 Fig — (A) The schematic illustrates the process of identifying pre-spatial domains that express positive markers and do not express negative markers within a 10 × 10 µm grid. (B) The schematic shows the workflow for identifying and stratifying the spatial domain using three sequential steps: median filtering, contouring, and measurement of distance. (C) The schematic demonstrates the procedure for extracting stratified spatial domains that do not overlap with other ones. (TIF) [file pcbi.1012854.s011.tif]

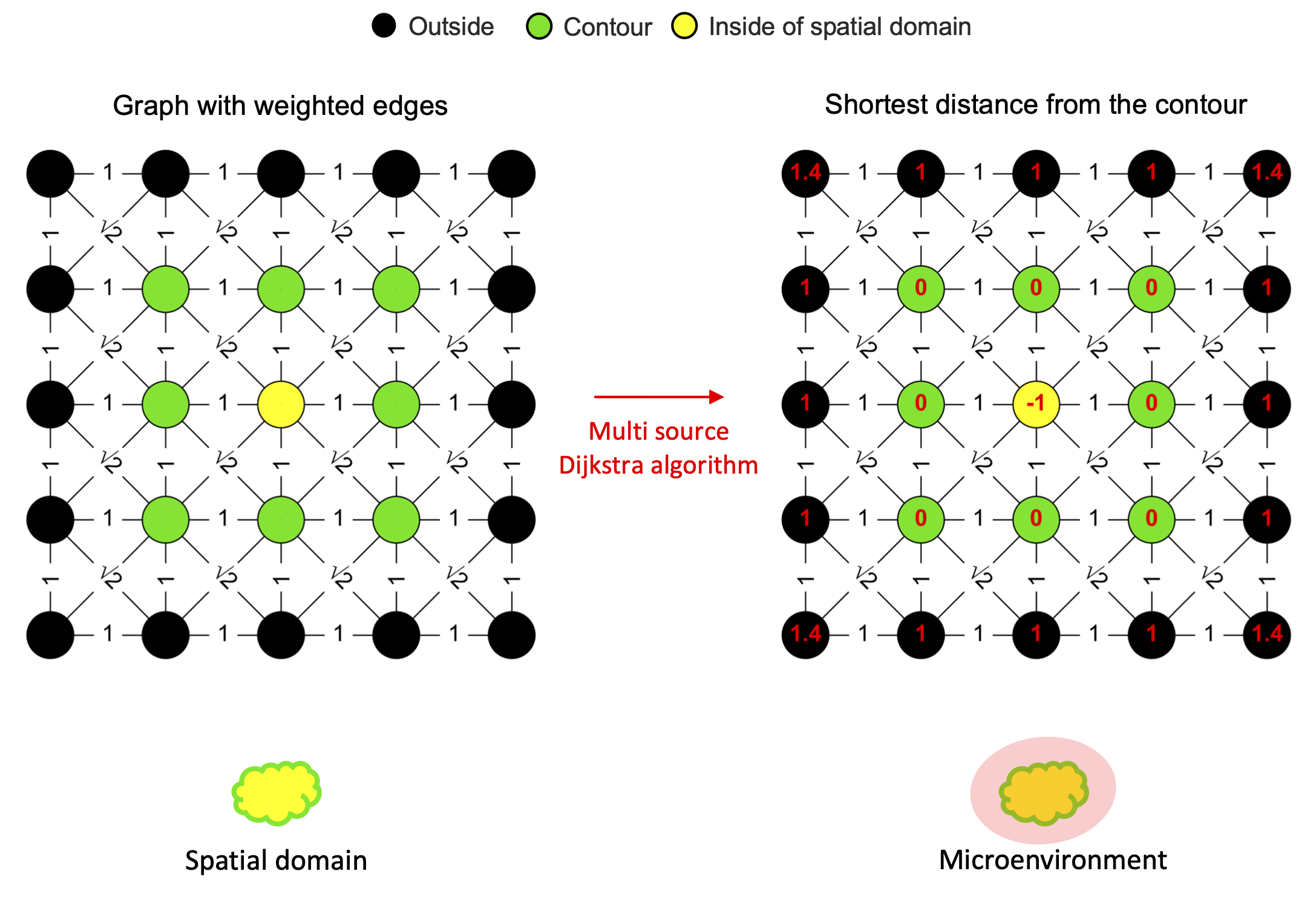

Supplement: S12 Fig — Left: Representation of a graph with weighted edges, where nodes are categorized as Outside the spatial domain (black), contour (green), and inside the spatial domain (yellow). Right: The result of applying the Multi-Source Dijkstra Algorithm is to compute the shortest distance from the contour. The calculated distances for each node are indicated in red. (TIF) [file pcbi.1012854.s012.tif]
