# Supplementary material for: SpatialKNifeY (SKNY): Extending from spatial domain to surrounding area to identify microenvironment features with single-cell spatial omics data
Source: PLoS Comput Biol. 2025 Feb 18;21(2):e1012854. doi: 10.1371/journal.pcbi.1012854 (PMC11849985; doi:10.1371/journal.pcbi.1012854)
Supplement: Attachment — Submitted filename: Response to Reviewers.docx [file pcbi.1012854.s013.docx]

Reviewer #1: The manuscript presents a toolkit that combines spatial omics data with histological image processing to study tumor microenvironments. With previously published breast and colorectal cancer datasets, SKNY detects spatial domains reflecting tumor regions, clusters these domains by malignancy, and extends analysis into the surrounding TME to assess cellular diversity and tumor progression trajectories. The study claims consistency with known cancer progression mechanisms and suggests SKNY’s capability for integrated analysis across patient samples to catalog TME features critical to disease progression. In my opinion, this manuscript is suited to be published in PLOS Computational Biology after addressing the following comments.

Response: We greatly appreciate your willingness to conduct our peer review. Below, we provide our responses to your point-by-point feedback.

1. In the introduction, it'd be helpful to elaborate on existing microenvironmental analysis tools in the context of spatial omics by pointing out their limitations and how SKNY solve them.

Response: Thank you for your valuable comments. We added the limitation of the current tools (STAGATE and Sopa) in the Introduction section to emphasize the novelty of SKNY (Page 5, Line 105-107).

**(Page 5, Line 105-107)**

Even though these tools are invaluable for extracting and characterizing spatial domains, they are limited in analyzing the surrounding area, particularly the microenvironment around the spatial domains.

2. The manuscript highlights increased diversity within spatial domains compared to isolated cancer cells, indicating the presence of various cell types within the TME. However, it lacks sufficient statistical explanation for how the α-diversity index directly correlates with spatial heterogeneity. A comparative analysis using other diversity indices or validation against independently annotated datasets would enhance credibility.

Response: Thank you for your helpful comments. We showed that gene expression was more diverse in the spatial domain than in the cellular domain for other indices, such as Shannon and the number of unique genes detected in each cell or spatial domain. We also confirmed the same trend for each cell type independently annotated by Janesick et al. (Page 9-10, Line 193-196, S4B-S4D Figs). We also added some sentences for them as below,

**(Page 9-10, Line 193-196)**

A similar trend was confirmed between the various cells independently annotated by Janesick *et al*. and the spatial domain by the three α-diversity indices of Chao1, observed features (the number of unique genes detected in each cell or spatial domain), and Shannon (S4B-S4D Figs).

**(S4 Fig)**

***S4 Fig. Comparison of alpha-diversity index based on gene expression.***

***(A)*** *Box plot of alpha-diversity index (Chao1) of expression between cancer cells and spatial domains. Box plot of* ***(B)*** *Chao1,* ***(C)*** *observed features, and* ***(D)*** *Shannon of expression among cells annotated by Janesick et al. and spatial domains.*

3. Clustering was conducted using PCA and UMAP, which provided interpretative 2D visualizations, yet no justification was given for the number of clusters. It would be beneficial to include a validation step, like silhouette analysis, to confirm the chosen number of clusters.

Response: Thank you for this comment. We performed silhouette analysis by changing the resolution of the Leiden algorithm parameters from 0.2 to 1.8 (Supplementary Figure, reviewers only). The results showed that the average silhouette value was highest when the resolution was 0.2, but one cluster did not reach the average silhouette value. For all other resolutions, the average silhouette values were around 0.5, and each cluster reached the average silhouette value. Based on these results, we chose the default values: resolution: 1.0, number of clusters: 9. The analysis involving this cluster could be explained from a biological perspective.

**(Supplementary Figure, reviewers only)**

***Supplementary Figure (reviewers only) Silhouette analysis for different cluster resolutions***

*The silhouette analysis evaluates the quality of clustering across varying resolutions (0.2–1.8) using the Leiden algorithm. Each subplot corresponds to a specific resolution, showing the silhouette coefficient distribution for all clusters. The red dashed line indicates the mean silhouette score for each resolution, reflecting the overall clustering quality.*

4. The study uses PAGA to model DCIS-to-IDC progression, but it could benefit from a pseudotime validation approach, such as Slingshot or Monocle, to ensure robust trajectory construction. Testing multiple trajectory algorithms would allow better triangulation of findings and validation of SKNY’s approach for trajectory estimation.

Response: We really appreciate this suggestion. We estimated the trajectory among spatial domains using Monocle and Slingshot and included the result on Page 12, Line 240-242, S5A and S5B Figs. These results reproduced the same pathway from the DCIS cluster to the IDC cluster via cluster 1, as in the PAGA algorithm.

**(Page 12, Line 240-242)**

Moreover, we confirmed that other algorithms, such as Monocle and Slingshot, estimated a similar trajectory (S5A and S5B Figs).

**(S5A and S5B Figs)**

***S5 Fig. Trajectory analysis of spatial domains using Monocle and Slingshot algorithms and autocorrelation analysis of marker gene for endothelial cell
(A)*** *The plot shows the Uniform Manifold Approximation and Projection (UMAP) representation of spatial domains, colored by their Leiden cluster assignments, overlaid with the trajectories predicted by the Monocle algorithm. The root point of the trajectory is labeled by the white node, and the branch points are labeled by the gray node.* ***(B)*** *The plot shows the UMAP representation of spatial domains overlaid by their Leiden cluster assignments (left) and overlaid with the trajectories predicted by the Slingshot algorithm (right).*

5. While significant associations between endothelial infiltration and cancer stages were identified, certain analyses lack sufficient statistical controls. The Kruskal-Wallis test is appropriate but limited for stratified spatial data. Applying mixed-effect models to account for intra-sample variability or leveraging spatial autocorrelation metrics could refine these results.

Response: Thank you for your important comments. To compare spatial characteristics, we calculated the spatial autocorrelation coefficient Geary's *C* within each cluster. We found that the DCIS-to-IDC cluster (Cluster 1) tended to have lower scores (higher autocorrelation) for the vascular endothelial cell marker genes (Page 14, Line 288-291, S5C Fig).

**(Page 14, Line 288-291)**

The spatial autocorrelation coefficient (Geary's *C*) of *PECAM1*, *VWF*, and *CD93* were calculated within each cluster, revealing that the DCIS-to-IDC cluster (Cluster 1) tended to have a lower score indicating higher autocorrelation (S5C Fig).

**(S5C Fig)**

***S5 Fig. Trajectory analysis of spatial domains using Monocle and Slingshot algorithms and autocorrelation analysis of marker gene for endothelial cell***

***(C)*** *Line plot depicting the relationship between Leiden clusters and Geary’s C for the genes PECAM1 (blue), VWF (orange), and CD93 (green).*

Reviewer #2: This paper presents SpatialKNifeY, that works on spatial transcriptomics data from Xenium platform. The tool can detect spatial domains and associated microenvironments. It can also provide trajectory estimation. The tool has been showcased on several datasets.

The code is well organized and documented in the Github repository. I have the following comments on the manuscript

Response: Thank you very much for taking on the peer review. We greatly appreciate your effort and time. We would like to provide our responses to your comments point by point as follows.

Line 68: Please use full form before using acronym

Response: Thank you for pointing this out. We added the 'tumor microenvironment' for TME (Page 4, Line 83).

**(Page 4, Line 83)**

Classical methods that examine the tumor microenvironment (TME) using a microscope capture histological features through staining or fluorescence-based technologies, leading to the discovery of pathological mechanisms in the microenvironment

I found the method difficult to understand (likely because of my confusion, as can be seen in comments that follow).

What is meant by pre-spatial domain? The authors need to clarify this.

Line 491: What is meant by pre-spatial domain?

Response: Thank you for your pointing this out. We have added a description and a Supplementary Figure (Page 26-27, Line 517-521, S11A Fig).

**(Page 26-27, Line 517-521)**

The pre-spatial domain ($S_{pre}$) was determined by subtracting the grids with a negative marker (example: *SFTPB*; breast cancer, melanoma, ovarian cancer, colorectal cancer, and metastatic colorectal cancer: N/A) from the grids with a positive marker (example and breast cancer: *CDH1*; melanoma: *MLANA*; ovarian cancer, colorectal cancer, and metastatic colorectal cancer: *EPCAM*) (S11A Fig).

**(S11A Fig)**

***S11Fig. Workflow of SpatialKNifeY algorithm***

***(A)*** *The schematic illustrates the process of identifying pre-spatial domains that express positive markers and do not express negative markers within a 10 × 10 µm grid.*

Line 499: I did not understand this equation. Perhaps, description will be helpful.

Response: Thanks again for pointing this out. We added a description in S11A Fig and its legend (Page 27, Line 522-530, S11 Fig, see above).

**(Page 27, Line 522-530)**

For example, if a user wants to obtain only the tumor region without the normal epithelium, logical subtraction between a positive marker's expression (e.g., *CDH1*) and a negative marker (e.g., *SFTPB*) can be performed.

$$S_{pre}=({Expr(Grid}_{column,row}^{positive marker})>0 )-(Expr({Grid}_{column,row}^{negative marker})>0)$$

where *Expr* is defined as a function of extracting gene expression counts from the grid (S11A Fig).

The STAGATE algorithm is misspelled as STARGATE in some places.

Response: Our apologies for the misspell. We made all the necessary corrections.

Line 513: What is meant by domain surface?
Line 514: What is an edge grid? Due to lack of my familiarity with this term, I was not able to follow this entire paragraph.

Response: We apologize for any confusion caused by our wording. We previously used the terms "domain surface" and "edge grid" interchangeably to refer to the “boundary line” of the spatial domain. We revised this terminology and added clarifying information in a supplementary figure (Page 28, Line 541-544, S11B Fig).

**(Page 28, Line 541-544)**

**Measurement of distance from the boundary line of the spatial domain**

The boundary line was identified using the ‘findContours’ function from opencv in the spatial domain (S11B Fig).

**(S11B Fig)**

***S11 Fig. Workflow of SpatialKNifeY algorithm***

***(B)*** *The schematic shows the workflow for identifying and stratifying the spatial domain using three sequential steps: median filtering, contouring, and measurement of distance.*

Line 516: it should be square root of 2, not 2. [It is correctly mentioned in the referred figure, however].

Response: Thank you for bringing the mistake to our attention. We have corrected it. (Page 28, Line 545)

**(Page 28, Line 545)**

All adjacent grids were connected by edges and weighted according to the Euclidean distance: 1 for vertical and horizontal edges and a root of 2 for diagonal edges

Line 522: It is not clear how connectedComponentsWithStats method from opencv could divide a spatial domain into individual spatial domains. The input needs to be an image. Do you pass in the entire image, or only a portion capturing the spatial domain?

Response: Thank you for your valuable comments. We integrated the entire image, including the spatial domain defined by findContours, into the connectedComponentsWithStats function and separated the spatial domain for each contour. The description was modified as follows: (Page 28, Line 552-554)

**(Page 28, Line 522-554)**

The function 'connectedComponentsWithStats' from opencv was used to divide the entire image of the spatial domain into individual spatial domain (*S*_d_, *d* = {1,2,3,…,426}).

I could not understand the notation in line 528, what is meant by P ?

Response: Thank you for your valuable comments. P (partial area) indicates the area separated from the spatial domain by 30 µm intervals; we modified the description referring to the Supplementary Figure (Page 29, Line 557-559, S11B Fig).

**(Page 29, Line 557-559)**

Using the measured distances (S12 Fig), a stratification was performed with a half-open interval of 30 µm to determine the partial area (*P*) as a stratified spatial domain ($P_{x<\mu\leq x+30}, x=\{-120,-90,-60,\ldots,150\}$) (S11B Fig).

**(S11B Fig)**

***S11 Fig. Workflow of SpatialKNifeY algorithm***

***(B)*** *The schematic shows the workflow for identifying and stratifying the spatial domain using three sequential steps: median filtering, contouring, and measurement of distance.*

I could not understand the equation in line 534. Why are there two union symbols? A written description would be beneficial here.

Response: Apologies for the error in the formula. It has been corrected as follows (Page 28-29, Line 489-508). To improve clarity, we have also included an explanation and added a Supplementary Figure (S11C Fig).

**(Page 29-30, Line 559-578)**

For the stratified spatial domain of the outer section of the spatial domain ($P_{x<\mu\leq x+30}, x=\{0,30,60,\ldots,150\}$), the rectangle that enclosed each $S_{d}$ was then extracted, and each rectangle was enlarged by *x* μm to produce a rectangle including each stratified spatial domain ($R_{d, x+30}, d=\left\{ 1,2,3,\ldots,426 \right\}, x=\left\{ 0,30,60,\ldots,150 \right\}$). The stratified spatial domain exclusive to the others (${PS}_{d, x<\mu\leq x+30}, d=\left\{ 1,2,3,\ldots,426 \right\}, x=\left\{ 0,30,60,\ldots,150 \right\}$) was calculated as follows:

$${PS}_{d, x<\mu\leq x+30}=P_{x<\mu\leq x+30} \bigwedge R_{d,x+30}\bigwedge\neg\bigcup_{0\leq i<j\leq n} (R_{i, x+30} \bigwedge R_{j, x+30})$$

where ⋀ represents the product set, and ⋃ represents the union set. The gene expression of each ${PS}_{d, x<\mu\leq x+30}$ was defined as the average gene expression of the grids within it. For simplicity, a flow assuming three adjacent spatial domains (*S*_1_, *S*_2_, and *S*_3_) and their (0, 30] stratified spatial domains (*P*_0<μ≤30_) is shown (S11C Fig). First, the rectangles covering the perimeter of each of *S_1_*, *S_2_*, and *S3* are extracted and expanded by 30 μm (*R*_1,30_, *R*_2,30_, and *R*_3,30_), and their pairwise intersection ($\bigcup_{0\leq i<j\leq n} (R_{i, x+30} \bigwedge R_{j, x+30})$) was taken. Then, to define stratified spatial domains specific to each spatial domain (*PS*_1, 0<μ≤30_, *PS*_2, 0<μ≤30_, and *PS*_3, 0<μ≤30_), *P*_0<μ≤30_, intersection sets of the rectangle (*R*_1_, *R*_2_, or *R*_3_), and complement of the pairwise intersection ($\neg\bigcup_{0\leq i<j\leq n} (R_{i, x+30} \bigwedge R_{j, x+30})$) were taken.

**(S11C Fig)**

***S11 Fig. Workflow of SpatialKNifeY algorithm***

***(C)*** *The schematic demonstrates the procedure for extracting stratified spatial domains that do not overlap with other ones.*

Line 554: typo snappy >> scanpy

Response: Thank you for pointing this out. We corrected it.

A flowchart of the method may be helpful.

Response: Thank you very much for your precise comment. We believe that the above S11 Fig will enhance the understanding of the analytical method.
